# Nature and Diffusion of Gynecologic Cancer–Related Misinformation on Social Media: Analysis of Tweets

**DOI:** 10.2196/11515

**Published:** 2018-10-16

**Authors:** Liang Chen, Xiaohui Wang, Tai-Quan Peng

**Affiliations:** 1 Lab for Big Data and Public Communication School of Communication and Design Sun Yat-sen University Guangzhou China; 2 Department of Journalism School of Communication Hong Kong Baptist University Hong Kong China (Hong Kong); 3 Department of Communication Michigan State University East Lansing, MI United States

**Keywords:** social media, breast cancer, cervical cancer, misinformation, diffusion, China

## Abstract

**Background:**

Over the last two decades, the incidence and mortality rates of gynecologic cancers have increased at a constant rate in China. Gynecologic cancers have become one of the most serious threats to women’s health in China. With the widespread use of social media, an increasing number of individuals have employed social media to produce, seek, and share cancer-related information. However, health information on social media is not always accurate. Health, and especially cancer-related, misinformation has been widely spread on social media, which can affect individuals’ attitudinal and behavioral responses to cancer.

**Objective:**

The aim of this study was to examine the nature and diffusion of gynecologic cancer–related misinformation on Weibo, the Chinese equivalent of Twitter.

**Methods:**

A total of 2691 tweets related to 2 gynecologic cancers—breast cancer and cervical cancer—posted on Weibo from June 2015 to June 2016 were extracted using the Python Web Crawler. Two medical school graduate students with expertise in gynecologic diseases were recruited to code the tweets to differentiate between true information and misinformation as well as to identify the types of falsehoods. The diffusion characteristics of gynecologic cancer–related misinformation were compared with those of the true information.

**Results:**

While most of the gynecologic cancer–related tweets provided medically accurate information, approximately 30% of them were found to contain misinformation. Furthermore, it was found that tweets about cancer treatment contained a higher percentage of misinformation than prevention-related tweets. Nevertheless, the prevention-related misinformation diffused significantly more broadly and deeply than true information on social media.

**Conclusions:**

The findings of this study suggest the need for controlling and reducing the cancer-related misinformation on social media with the efforts from both service providers and medical professionals. More specifically, it is important to correct falsehoods related to the prevention of gynecologic cancers on social media and increase individuals’ capacity to assess the veracity of Web-based information to curb the spread and thus minimize the consequences of cancer-related misinformation.

## Introduction

### Background

In recent years, cancer has become a major public health issue in China. According to the statistics from the National Central Cancer Registry, there were approximately 3.80 million new cancer cases and 2.30 million cancer-caused deaths in China in 2014 [1]. For women, 2 out of the 10 most common cancers are gynecologic cancers, with breast cancer (268,600 new cases) and cervical cancer (98,900 new cases) being the most prevalent in 2015 [2]. The incidence and mortality rates of gynecologic cancers have increased substantially in China over the last 2 decades, and it has been 1 of the major health concerns for women in China [2].

With the rapid development of social media, social media has become a popular means by which individuals can access a staggering amount of health information [3,4]. A growing number of individuals, especially women, turn to social media to seek out and share a variety of cancer-related information, such as seeking information about cancer prevention and treatment as well as sharing the experience of having it, and to obtain social support to cope with the disease and manage emotions [5,6]. Medical professionals and traditional portals contribute to the health information available on social media, but a greater amount of information is generated and disseminated by ordinary users based on their first-hand cancer experiences [7]. Web-based health information has been found to be effective in raising individuals’ awareness of diseases and fueling communication between lay persons and health care professionals [8]. Furthermore, Web-based health information could help individuals improve their abilities to prevent certain diseases and enable them to effectively manage chronic health conditions [9,10].

Nevertheless, individuals might take great risks in their utilization of Web-based resources, as health information on social media is not always accurate [11,12]. It has been shown that health-related misinformation in general, and cancer-related misinformation in particular, has been widely spread on social media, which affects individuals’ responses to cancer prevention and treatment [13]. Moreover, because of overloaded information on social media, ordinary users may not have the resources, knowledge, and expertise to assess the veracity of Web-based cancer-related information and to identify informative and trustworthy information on social media [13].

Although scholarly attention has been drawn to misinformation on social media, little is known about the nature and diffusion of cancer-related misinformation there. In this study, we filled this gap by examining the nature and diffusion of misinformation about breast cancer and cervical cancer on social media. Specifically, we used a content analysis not only to differentiate between true information and misinformation regarding these 2 types of gynecologic cancers on social media but also to identify the types of falsehoods in such information. Furthermore, the diffusion characteristics of cancer-related misinformation were examined and compared with those of true information.

### Misinformation on Social Media

Misinformation refers to false and inaccurate information that is spread intentionally or unintentionally [14]. With the increasing penetration of information and communication technologies, massive amounts of misinformation can be easily disseminated to a larger group of audience at very low costs. One study revealed that on average, individuals in the United States encountered 1 to 3 fake news stories online in the month before the 2016 US election [15]. About one-quarter of adults reported having shared fabricated political news online, sometimes by mistake and sometimes intentionally [16].

Many scholars have argued that social media is responsible for the high prevalence of Web-based misinformation [17]. Traditional media content is usually produced by professional journalists and editors who are information gatekeepers with adequate knowledge and resources to assess the veracity of information. However, ordinary users of social media are empowered to produce and share a wide variety of information irrespective of its veracity [18]. Thus, it is not surprising that there is a large amount of misinformation on social media.

The proliferation of Web-based misinformation has caused negative consequences for both individuals and the society as a whole. Specifically, misinformation, such as fake news, rumors, and inaccurate information, not only causes the spread of unnecessary fears and conspiracies but also distorts individuals’ behavioral responses to certain issues, such as political elections, natural disasters, and diseases [16,19]. For example, misinformation about vaccinations makes many parents refuse immunizations for their children, which has led to a noticeable increase in vaccine-preventable diseases and has even caused deaths among children [20]. Furthermore, misinformation exerts negative impacts on our society that may trigger financial panic and even strain diplomatic relations [21]. The 2013 World Economic Forum listed misinformation as one of the main threats to human society [17].

To constrain the amount of misinformation spread on social media and to minimize the negative effects caused by the misinformation, many researchers have attempted to examine how misinformation spreads on social media and investigate the driving mechanisms that underlie the diffusion of Web-based misinformation in various domains, such as natural disasters, science, and politics. Specifically, Oh et al [22] analyzed the working dynamics of rumors related to the Haiti earthquake in 2010 based on data from Twitter and found that informational uncertainty and anxiety are key factors that determine the rapid spread of a rumor. Moreover, they indicated that reliable information with credible sources could reduce levels of anxiety on Twitter, which in turn limits the spread of rumors. Domenico et al [23] explored the spread of a scientific rumor about the Higgs boson and proposed a model for its spread. They found that individuals were more likely to spread the rumor if most of their friends tweet it repeatedly. More recently, Vosoughi et al [19] explored the diffusion structure of true and false news on Twitter and found that false news spread faster, deeper, and more broadly than the truth. Such differences in the diffusion of truth and falsehoods may be related to the fact that false news tends to include more emotion of fear, disgust, and surprise, which could cause the misinformation to go viral. Another explanation could be a novelty effect, that is, people are more willing to exchange novel information [19]. A study by Zhao et al [24] is one of the few studies that had investigated the misinformation on Chinese social media. They indicated that when large-scale social crises occur, a great number of rumors are posted and reposted quickly on social media. Moreover, they found that attitude and personal norms are the key factors, which would drive social media users in China to combat rumors.

Although much attention has been directed toward the spread of misinformation on social media, most studies have focused on political or scientific misinformation in general. Only a few studies have examined the nature and diffusion of health, especially cancer-related misinformation on social media. The uniqueness in the context of cancer-related information requires a specific investigation.

### Cancer-Related Misinformation on Social Media

The rise of news media has created an atmosphere of hype and hysteria about cancer in which individuals have been exposed to conflicting information [25]. This leads to many misperceptions about cancer, including its causes, prevention, and treatment [25]. In the last decade, social media has exacerbated individuals’ uncertainty about cancer. Unlike traditional media, most health-related content on social media is generated and shared by patients and caregivers based on their own personal experiences. The content may include many false elements that can distort individuals’ attitudes and behaviors toward cancer prevention and treatment. Gage-Bouchard et al [26] conducted a content analysis to assess the veracity of information related to lymphoblastic leukemia on 19 public Facebook pages and found that at least one-third of the exchanged information was not medically or scientifically accurate.

With its extremely large population, China contributes significantly to the global burden of cancer [27]. Gynecologic cancers (eg, breast cancer, ovarian cancer, and cervical cancer) have become the most common cancers among Chinese women [2]. However, cancer is preventable if people are aware of its causes and science-based prevention strategies [28]. As more and more female users in China have used social media to exchange a variety of cancer-related information [6,26,29], this study focused on misinformation regarding breast cancer and cervical cancer on Chinese social media.

### Research Questions

On the basis of the aforementioned literature, misinformation, especially health misinformation, has been prevalent on social media, which has drawn much attention from the governments, academia, and industry [13]. Recently, several studies have examined the veracity of cancer-related information on social media. However, most of these studies were exploratory in nature, only describing the prevalence of misinformation, and did not consider the types of misinformation and their diffusion characteristics [26]. To fill in the gaps in the literature, this study focuses on 2 gynecologic cancers, namely breast and cervical cancers, and proposes the following 3 research questions:

- Research question 1: What is the distribution of true information and misinformation regarding gynecologic cancers on Weibo, a Chinese version of Twitter?
- Research question 2: What kinds of gynecologic cancer–related misinformation exist on Weibo?
- Research question 3: How do the diffusion characteristics of true information and misinformation regarding gynecologic cancers differ?

To answer our research questions, a content analysis was first conducted to differentiate between true information and misinformation and to identify the types of falsehoods embedded in the misinformation. Next, the diffusion structure of each piece of cancer-related information on social media was constructed and analyzed with a network perspective to understand how misinformation was spread and received by social media users.

## Methods

### Data Collection

Two keywords “乳癌/乳腺癌” [breast cancer] and “子宫癌/宫颈癌” [cervical cancer] were employed to search tweets about breast cancer and cervical cancer on Weibo, one of the most popular social media platforms in China. We randomly selected 7 weeks out of 52 weeks from June 2015 to May 2016. Tweets posted in the 7 weeks were retrieved and included in the study. In total, 2691 tweets were extracted with the Python Web Crawler. The content, post time, and diffusion path of each tweet were retrieved.

In terms of ethical issues, Weibo is considered a public domain in which data are freely accessible to the public. To minimize the potential harm to Weibo users, all the data collected from Weibo were deindividualized to maintain the users’ anonymity. Moreover, all of the tweets presented in this paper were paraphrased or written in aggregate to prevent identification of the users.

### Coding Procedure

A total of 2 medical school graduate students with expertise in gynecologic diseases were recruited to complete the coding. Initially, the 2 coders were asked to pilot the project by coding 10.41% (280/2691) of the total tweets to develop and refine the coding schemes. Of the 2691 total tweets on Weibo, 1144 tweets (1144/2691, 42.51%) only expressed personal emotions and experiences that cannot be identified as truth or falsehood. These tweets were excluded from further analysis. The remaining 1547 (1547/2691, 57.49%) tweets contained medically oriented information for which the thematic category and information veracity were coded.

First, 4 *thematic categories* of all the 1547 tweets were coded: (1) background knowledge, which refers to basic information about breast cancer and cervical cancer, including the prevalence, causes, and symptoms of each cancer; (2) prevention, which refers to methods and actions that can lower the risk of getting the cancer under study, includes maintaining a healthy lifestyle, avoiding exposure to known cancer-causing substances, and taking medicines or vaccines; (3) diagnosis, which refers to the act of identifying a disease from its signs and symptoms; and (4) treatment, which refers to drugs or methods that can attack specific types of cancer cells to help the patient fight the disease. These 4 thematic categories are mutually exclusive, implying that each tweet will be assigned to 1 theme only. Krippendorff alpha [30] for this round coding was .95, which means that the intercoder reliability for thematic category is well accepted.

Second, the 2 coders coded the *information veracity* of the 1547 tweets. Specifically, the coders categorized each tweet as 1 (=true information) and 2 (=misinformation) for the tweets in the 4 thematic categories. Krippendorff alpha tests [30] revealed an acceptable level of intercoder reliability for all of the variables: .91 for background knowledge, .88 for prevention, .93 for diagnosis, and .89 for treatment.

Finally, when a tweet was categorized as *misinformation* in each thematic category, the 2 coders indicated the types of falsehoods using a conventional content analysis. Conventional content analysis is a qualitative approach widely used in health research [31]. First, all of the false tweets were read repeatedly to achieve immersion and obtain a sense of the entire situation [32]. Second, while reading the tweets, the 2 coders highlighted exact words from the text as codes or created new codes to capture key concepts [33]. Third, these codes were sorted into categories based on their relationships. Thereafter, the second and third procedures were repeated to keep the acceptability and reliability of the designated categories high. Finally, each category was defined. The validity of the coding was checked using a deviant case analysis.

### Quantifying the Diffusion Characteristics of Gynecologic Cancer–Related Information

The diffusion characteristics of all the 1547 tweets were measured with following 5 characteristics: the scale of retweets, the range of retweets, the structural virality of retweets, the number of comments, and the number of likes. These 5 indices measure the diffusion breadth, diffusion depth, and the engagement of the information relevant to breast and cervical cancers [31].

The retweet network of all 1547 tweets was first constructed by tracking how each original tweet was retweeted. The scale, range, and structural virality of the retweet networks were estimated and assigned as diffusion indices for each tweet. The scale of retweets is the number of total retweets received by a tweet. The range of retweets refers to the depth of a retweet network as indicated by the number of hops in a diffusion chain [34]. The structural virality of retweets measures the divergent branches in the diffusion network [35], which is equal to the average distance between all pairs of nodes in a retweet network.

Beyond the characteristics derived from the retweet networks, comments and likes received by a tweet can represent users’ engagement in the process of information spreading [36]. Thus, the number of comments and the number of likes received by each tweet are included as the other 2 diffusion characteristics of the gynecologic cancer–related information in the study. The 5 characteristics capture the information diffusion on social media from a multidimensional perspective, which provides a more comprehensive understanding of the diffusion structures of Web-based information.

## Results

### Nature of Gynecologic Cancer–Related True Information and Misinformation

Among the 1547 medically oriented tweets, the most commonly exchanged type of cancer-related information was background knowledge (749/1547, 48.42%), followed by prevention (467/1547, 30.19%), treatment (189/1547, 12.21%), and diagnosis (142/1547, 9.18%). Moreover, 66.13% (1023/1547) of the tweets provided true information and 33.87% (524/1547) contained misinformation. A chi-square test indicated that true information was significantly more prevalent than misinformation, χ^*2*^_1(N=1547)_=160.9, *P*<.001.

Information in 4 thematic categories was found to differ significantly on their information veracity, χ^*2*^_3(N=1547)_=322.5, *P*<.001. Tweets on treatment contained a higher percentage of misinformation than true information; specifically, 156 (156/189, 82.5%) tweets related to cancer treatment included misinformation. These were followed by tweets about background knowledge; of these, 287 (287/749, 38.3%) tweets contained misinformation. Only 14.4% (67/467) of prevention-related tweets and 14 (14/142, 9.9%) diagnosis-related tweets were not medically accurate (see Table 1).

The types of falsehoods were identified for information in each thematic category as summarized in Table 2. Specifically, the falsehoods in the category of background knowledge mainly included epidemiology, risk factors, prognosis, and pathology. Prevention-related tweets had a relatively small amount of misinformation that involved 2 types of falsehoods: lifestyle and vaccinations. Diagnosis-related misinformation was divided into 2 types: clinical manifestations and diagnostic techniques. Cancer treatment–related misinformation mainly included surgery, radiation therapy, drug therapy, and other therapies.

**Table 1:** Distribution of gynecologic cancer–related information by thematic category and information veracity.

Information veracity	Thematic category			
	Background knowledge, n (%)	Prevention, n (%)	Diagnosis, n (%)	Treatment, n (%)
True information	462 (61.7)	400 (85.7)	128 (90.1)	33 (17.5)
Misinformation	287 (38.3)	67 (14.4)	14 (9.9)	156 (82.5)
Total	749 (100.0)	467 (100.0)	142 (100.0)	189 (100.0)

**Table 2:** Types of falsehoods in different thematic categories of gynecologic cancer–related information on Weibo.

Thematic category and types of falsehoods		Definition	Example
**Background knowledge**			
	Epidemiology	The distribution and determinants of health and disease conditions in specified populations	The cancer prevalence rate is 10% higher in China than the world average
	Risk factors	An aspect of personal behavior or lifestyle, environmental exposure, inborn or inherited characteristic, which on the basis of epidemiological evidence, is known to be associated with a health-related condition	Using preservative-containing cosmetics is one of the main causes of breast cancer
	Pathology	A specialty concerned with the nature and cause of disease as expressed by changes in cellular or tissue structure and function caused by the disease process	Breast hyperplasia is the beginning of breast cancer
	Prognosis	A prediction of the probable outcome of a disease based on an individual’s condition and the usual course of the disease as seen in similar situations	Triple-negative breast cancer has a better prognosis than the normal type, and the 5-year survival rate is high
**Prevention**			
	Lifestyle	Typical way of life or manner of living characteristic of an individual or group	Drinking 5 cups of coffee a day or regular exercise, such as cycling, could reduce the risk of developing breast cancer by at least 20%
	Vaccinations	Administration of vaccines to stimulate individuals’ immune responses	The HPV^a^ vaccine can reduce the risk of cervical cancer by 100%
**Diagnosis**			
	Clinical manifestations	A symptom is observed by the patient subjectively but cannot be measured directly, whereas a sign is objectively observable by others	Any abnormality of the breast is an early symptom of breast cancer
	Diagnostic techniques and procedures	Methods, procedures, and tests performed to diagnose a disease, disordered function, or disability	A compound derived from urinary thiol is the only reagent that can detect early cervical cancer
**Treatment**			
	Surgery	Operations conducted for the correction of deformities and defects, repair of injuries, and diagnosis and cure of certain diseases	Precancerous lesions in the endometrium indicate the need for surgery to remove the uterus
	Radiotherapy	Ionizing radiation conducted to treat malignant neoplasms and some benign conditions	Up to 60% of cancer patients need radiotherapy in various stages of treatment
	Drug therapy	Drugs and chemicals, including chemotherapy, targeted therapy, and endocrine therapy	The new drug pertuzumab (Perjeta) has been used together with herceptin and chemotherapy to shrink tumors completely, so some patients do not need surgery
	Other therapies	Other therapies, including traditional Chinese medicine, biotherapy, and interventional therapy	With the application of traditional Chinese medicine, most patients with breast cancer will not need surgery

^a^HPV: human papillomavirus.

### Diffusion Characteristics of Gynecologic Cancer–Related Information

Among all of the 524 tweets categorized as misinformation, only 64 received retweet or retweets (mean 5.09 [SD 70.40]), 72 received comment or comments (mean 0.53 [SD 2.62]), and 132 received like or likes (mean 2.68 [SD 31.80]). The popularity of misinformation in terms of its diffusion was unevenly distributed, with several tweets receiving a large number of retweets, whereas the majority received no retweets or likes. For instance, the 2 most popular false tweets were about gynecologic cancer prevention and treatment methods that involved eating specific foods, such as garlic, mushrooms, and red wine; these received 1143 and 1131 retweets, respectively.

**Figure 1:**
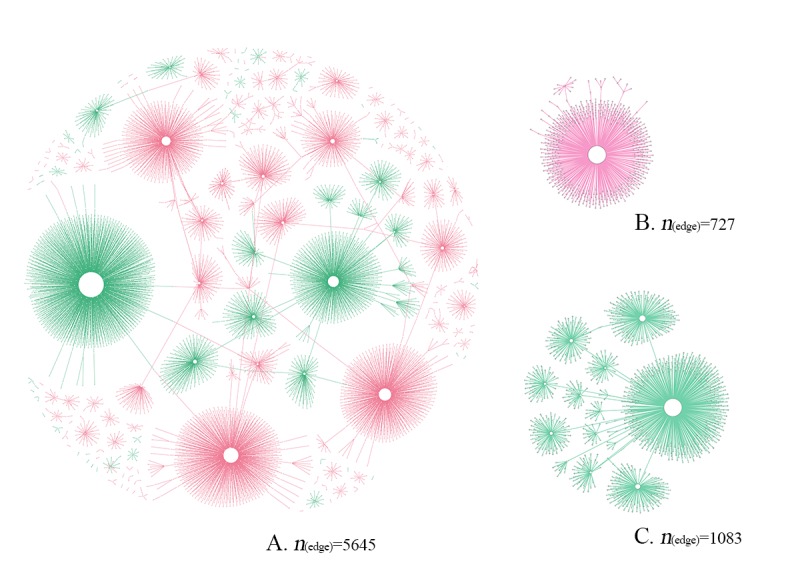
An illustration of the retweet network: (A) the full retweet network of all true information (red) and misinformation (green); (B) the largest retweet network of true information; (C) the largest retweet network of misinformation.

Among the 1023 tweets categorized as true information, only 143 received retweet or retweets (mean 4.06 [SD 32.58]). A total of 120 tweets received comment or comments (mean 1.78 [SD 14.41]), and 167 tweets received like or likes (mean 2.54 [SD 28.08]). Similarly, most of the tweets vanished into obscurity after being published, and several tweets reached a high degree of popularity. Moreover, most of these popular tweets were about cancer prevention methods, such as lifestyle and vaccinations. Figure 1 displays the retweet network of all the cancer-related true information and misinformation as well as the largest retweet networks for both true information and misinformation.

**Figure 2:**
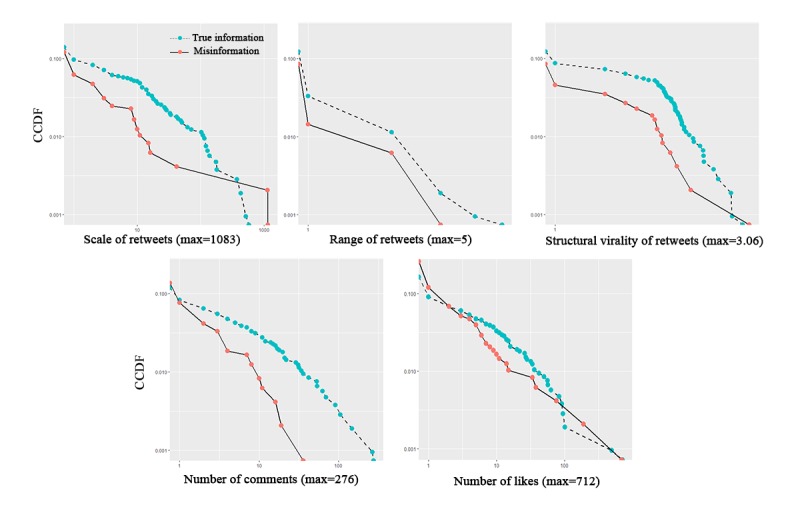
Complementary cumulative distribution functions of true information and misinformation cascades (x-axis and y-axis are log-transformed).

By comparing the diffusion characteristics of the true information and misinformation, it was found that true information was generally better diffused and accepted than misinformation by social media users. Figure 2 shows the complementary cumulative distribution functions of the 5 diffusion characteristics of true information and misinformation. Although several false tweets had been extremely popular and received a large number of retweets, most of the false tweets received less retweets than the true tweets. In addition, true tweets had better diffusion performance in terms of the range and structural virality of retweet networks and the number of comments. All of the indices showed that true information spread more deeply and broadly than misinformation, reaching a larger audience on social media.

A between-subject multivariate analysis of variance (MANOVA) was performed to test the differences on the 5 diffusion characteristics between information in different thematic categories and those with different information veracity. Information in the diagnosis category was excluded in the analysis, as there were not adequate cases in the misinformation group (n=14).

**Figure 3:**
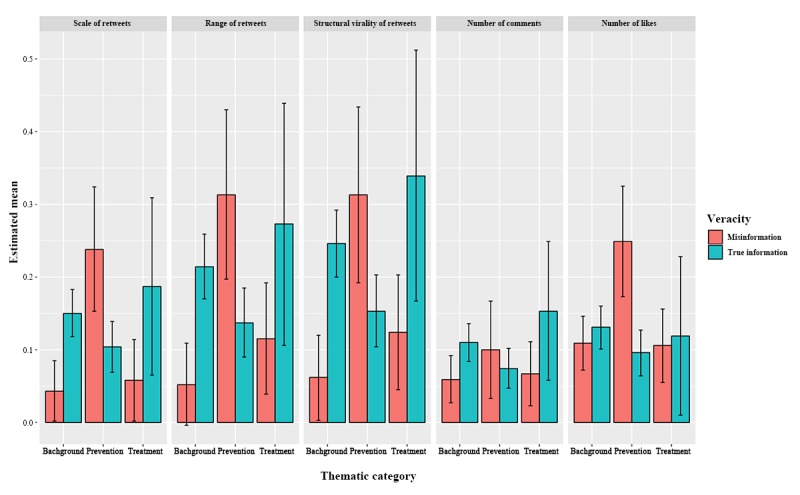
Estimated diffusion characteristics adjusted by thematic category and information veracity. Means reported here are estimated marginal means of multivariate analysis of variance (MANOVA). Outliers, multivariate normality, linear relationships between dependent variables, and multicollinearity were checked before analysis. Scale of retweets, number of comments, number of likes were log-transformed to fit the assumption of normal distribution.

The MANOVA results show that the interaction effect between thematic category and information veracity was significant (*F*_2,1399_=3.26, *P*<.001; Wilks lambda=0.98;*η* p^2^=.011). Figure 3 reports the estimated means of 5 diffusion characteristics adjusted by information veracity and thematic category, as well as their CI at 95% confidence level.

Specifically, regarding the thematic category of background knowledge, there were significant differences between the true information and misinformation groups in the scale, range, and structural virality of retweet networks, although there were nonsignificant differences in the number of likes and number of comments. It showed that individuals were more likely to spread true information about background knowledge than misinformation.

In terms of the thematic category of treatment, although estimated means revealed that true information generally had higher diffusion indices than misinformation, these differences were not significant. In addition, for prevention-related information, significant differences between the true information and misinformation groups occurred in the scale and range of retweets as well as the number of likes. There was no significant difference in the number of comments and structural virality of retweets. According to Figure 3, prevention-related misinformation spread better than true information on social media.

## Discussion

### Principal Findings

Individuals have increasingly used social media to exchange cancer-related information[5,26]. However, such information may includemany false elements, and these could distort individuals’ attitudes and behaviors toward cancer prevention and treatment. This study seeks to understand thenature and diffusion of cancer-related misinformation on social media.

First, the findings revealed that of the 2691 total tweets examined, more than half included medically oriented information about cancer. Although most of the medically oriented tweets provided accurate information, more than 30% contained misinformation. This finding suggests that the public should assess the information veracity of tweets on social media before accepting and following the advice embedded in such information. Moreover, the large amount of cancer-related misinformation on social media suggests the need for correction and reduction of misinformation, with the efforts from both social media service providers and medical professionals. For instance, with the knowledge from medical professionals, social media service providers could establish a digital library for cancer-related misinformation, which could provide a scaffold for self-checking by the public [37].

In addition, the results indicated that social media tweets related to cancer treatment contained a substantially greater percentage of misinformation than true information. However, the network analysis of the information diffusions showed that cancer treatment–related misinformation did not outperform the true information in terms of their diffusion characteristics. Moreover, for background knowledge, true information spread to a wider range of audiences than misinformation. These findings were inconsistent with those of Vosoughi et al’s study [19] in which misinformation diffused better than true information. The inconsistency may be caused by the unique context of gynecologic cancers. Unlike Vosoughi et al [19], who examined all types of news stories on Twitter, we focused on misinformation related to 2 specific types of gynecologic cancers only. Due to the wide media coverage of female celebrities diagnosed with breast and cervical cancers, many Chinese people are well aware of the topic [38], thus limiting the spread of misinformation related to it on social media. This implies that future research on the spread of misinformation on Web should adopt a topic-specific or domain-specific approach.

More interestingly, the diffusion characteristics of prevention-related misinformation are quite different from the information in other thematic categories. Although there was a relatively small amount of prevention-related misinformation on social media, this misinformation diffused significantly more broadly and deeply than true information. One possible explanation could be that a large amount of prevention-related misinformation provided ways or actions to prevent breast cancer and cervical cancer that individuals could perform by themselves. In other words, the prevention-related misinformation contained both self-efficacy and response efficacy, which could help individuals reduce anxiety and fear as well as control their perceived threat from cancer [39]. Thus, individuals are more willing to spread these prevention-related messages.

### Implications

Several practical implications can be derived here. First, medical professionals should make efforts to correct misinformation regarding the appropriate ways of preventing gynecologic cancers and decrease the spread of cancer-preventing misinformation on social media. This calls for the establishment of online health communities to list common cancer-related misinformation and provide accurate information about cancer prevention to address the public misperceptions of cancer [40]. Second, the government should run health campaigns and education programs to improve the public’s health literacy and strengthen their capacities to obtain, read, understand, and assess health care information so that they can use Web-based health information effectively and make appropriate health decisions [41,42]. Third, the public should be encouraged to verify the accuracy of Web-based cancer-related information, especially preventive information such as superfoods and vaccinations, to protect them from using counterfeit, inappropriate, or unsafe cancer prevention measures, as suggested by Bode and Vraga (2018) [43]. Finally, the significant difference between the diffusion characteristics of true information and misinformation implies that those diffusion characteristics can act as heuristics to identify cancer misinformation. In other words, the spread of misinformation on Web usually follows specific patterns that are different from true information, suggesting that interventions can come into play in the early stage of misinformation diffusion [19].

### Limitations

Several limitations of this study should be acknowledged. First, this study only focused on gynecologic cancer–related information. The public’s awareness and knowledge about specific diseases and health issues may vary to a different extent, which might affect how they perceive and disseminate relevant misinformation on social media. Thus, future studies could examine the nature and diffusion of misinformation regarding other disease or health issues in different cultural settings.

Second, although this study provided an empirical investigation on the types of gynecologic cancer–related misinformation and its diffusion characteristics, the factors driving the diffusion of different types of misinformation remain unknown. Future research should examine the mechanisms behind the diffusion of misinformation on social media and elucidate effective strategies for curbing the spread of misinformation. Finally, the data were extracted in July 2016, which were 2 years old. With the increasing popularity of social media, people’s health literacy has been continuously improving over the last few years. They are increasingly cautious about posting and sharing health information on social media, which might change the nature and diffusion of cancer information. Thus, future research with a wider time span could be conducted to investigate the inherent changes of cancer information diffusion on social media.

### Conclusions

This study makes the first attempt to examine the nature and diffusion of cancer-related misinformation on Chinese social media. First, the gynecologic cancer–related tweets were content-analyzed to differentiate between true information and misinformation on social media as well as to identify the types of falsehoods. In addition, a network perspective was adopted to examine the diffusion characteristics of misinformation through comparisons with those of true information. The results indicated that although most of the gynecologic cancer–related tweets provided medically accurate information, approximately 30% contained misinformation.

More importantly, although cancer treatment–related tweets included a great amount of misinformation, the misinformation did not diffuse significantly greater than true information. Conversely, cancer prevention tweets contained a relatively small amount of misinformation, but it spread more broadly and deeply than true information. These findings suggest that the government, social media service providers, and medical professionals should make great efforts to decrease the prevalence of cancer misinformation on social media. Moreover, health campaigns and programs should be conducted to increase the public’s motivations and abilities to verify Web-based cancer-related information, especially preventive measures before sharing or following the instructions from these messages.

## Abbreviations

MANOVA: multivariate analysis of variance
